# (2*S*,4*S*)-3-Acryloyl-6-oxo-2-phenyl­perhydro­pyrimidine-4-carboxylic acid

**DOI:** 10.1107/S1600536809050892

**Published:** 2009-12-04

**Authors:** Sandip K. Kundu, Mathew P. D. Mahindaratne, Brian Quinõnes, George R. Negrete, Edward R. T. Tiekink

**Affiliations:** a Department of Chemistry, The University of Texas at San Antonio, One UTSA Circle, San Antonio, Texas 78249-0698, USA; b Department of Chemistry, University of Malaya, 50603 Kuala Lumpur, Malaysia

## Abstract

In the title compound, C_14_H_14_N_2_O_4_, the central six-membered ring adopts a twisted boat conformation with the phenyl substituent occupying an orthogonal position [dihedral angle = 86.88 (11)°]. In the crystal, mol­ecules are linked by carboxylic acid–carbonyl O—H⋯O and amide–carbonyl N—H⋯O hydrogen bonds, forming a three-dimensional network.

## Related literature

For the synthesis from (*S*)-asparagine, see: Lakner & Negrete (2002 ▶). For background to water-soluble chiral auxiliaries, see: Mahindaratne *et al.* (2005*a* ▶,*b* ▶). For conformational analysis, see: Cremer & Pople (1975 ▶).
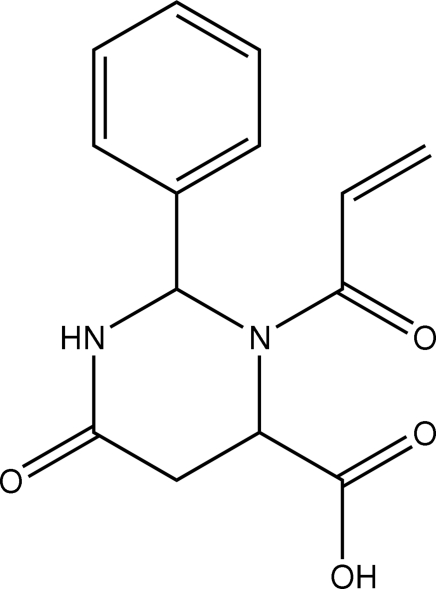


## Experimental

### 

#### Crystal data

C_14_H_14_N_2_O_4_
*M* *_r_* = 274.27
Orthorhombic, 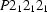

*a* = 10.573 (5) Å
*b* = 10.670 (7) Å
*c* = 11.612 (5) Å
*V* = 1310.0 (12) Å^3^
*Z* = 4
Mo *K*α radiation
μ = 0.10 mm^−1^
*T* = 98 K
0.36 × 0.12 × 0.12 mm

#### Data collection

Rigaku AFC12/SATURN724 diffractometer
Absorption correction: multi-scan (*ABSCOR*; Higashi, 1995 ▶) *T*_min_ = 0.496, *T*_max_ = 1
14053 measured reflections
1573 independent reflections
1547 reflections with *I* > 2σ(*I*)
*R*_int_ = 0.033

#### Refinement

*R*[*F* ^2^ > 2σ(*F* ^2^)] = 0.038
*wR*(*F* ^2^) = 0.097
*S* = 1.11
1573 reflections
185 parameters
1 restraint
H-atom parameters constrained
Δρ_max_ = 0.20 e Å^−3^
Δρ_min_ = −0.20 e Å^−3^

### 

Data collection: *CrystalClear* (Rigaku/MSC, 2005 ▶); cell refinement: *CrystalClear*; data reduction: *CrystalClear*; program(s) used to solve structure: *SHELXS97* (Sheldrick, 2008 ▶); program(s) used to refine structure: *SHELXL97* (Sheldrick, 2008 ▶); molecular graphics: *DIAMOND* (Brandenburg, 2006 ▶); software used to prepare material for publication: *publCIF* (Westrip, 2009 ▶).

## Supplementary Material

Crystal structure: contains datablocks global, I. DOI: 10.1107/S1600536809050892/hg2611sup1.cif

Structure factors: contains datablocks I. DOI: 10.1107/S1600536809050892/hg2611Isup2.hkl

Additional supplementary materials:  crystallographic information; 3D view; checkCIF report

## Figures and Tables

**Table 1:** Hydrogen-bond geometry (Å, °)

*D*—H⋯*A*	*D*—H	H⋯*A*	*D*⋯*A*	*D*—H⋯*A*
N1—H1⋯O41^i^	0.88	2.05	2.812 (3)	144
O42—H42*o*⋯O6^ii^	0.84	1.76	2.596 (3)	173

Symmetry codes: (i) 
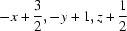
; (ii) 
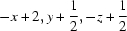
.

**Fig. 1.**
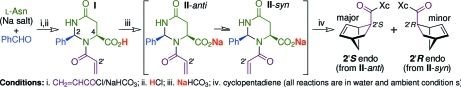
Reaction scheme.

**Fig. 2.**
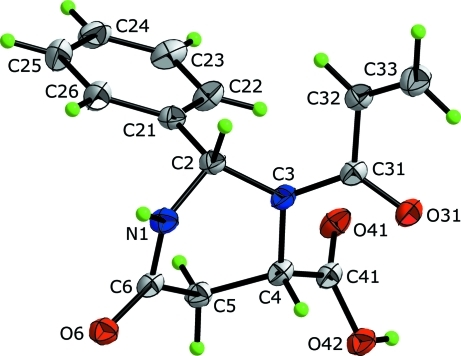
Molecular structure of (I), showing atom-labelling scheme and displacement ellipsoids at the 50% probability level.

**Fig. 3.**
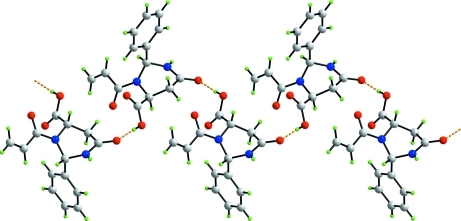
Supramolecular chain aligned along the b axis mediated by O–H···O hydrogen bonds (orange dashed lines). Color code: oxygen, red; nitrogen, blue; carbon, grey; and hydrogen, green.

**Fig. 4.**
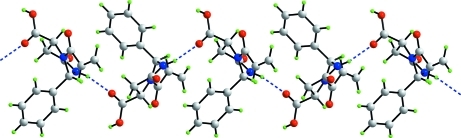
Supramolecular chain aligned along the c axis mediated by N–H···O hydrogen bonds (blue dashed lines). Color code: oxygen, red; nitrogen, blue; carbon, grey; and hydrogen, green.

**Fig. 5.**
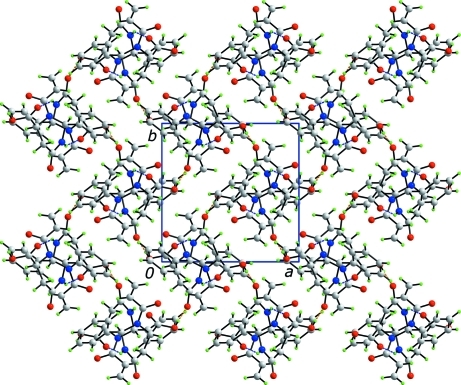
Unit-cell contents viewed in projection down the c axis. The O–H···O and N–H···O hydrogen bonds are shown as orange and blue dashed lines, respectively.

## supplementary crystallographic information

### Comment

The title compound (I), was prepared as a part of an on-going program aimed at developing water-soluble chiral auxiliaries (Mahindaratne *et al.*, 2005a,b). The development of effective, water-soluble chiral auxiliaries is attractive from an environmental standpoint. We have been investigating the use of asparagine-derived auxiliaries for asymmetry transfer in Diels-Alder cycloadditions under aqueous conditions. Herein, we report the structure of the derivative (I), which was prepared upon cyclocondensation of *L*-asparagine under basic conditions with benzaldehyde followed by *in situ* acryloylation (Fig. 1). Precipitation occurred on addition of HCl and washing the precipitate with cold water yielded analytically pure acrylamide, (I).

The central ring in (I) adopts a twisted boat conformation, Fig. 2, whereby the RMS of the N1, N3, C4 and C6 atom is 0.057 Å with the C2 and C5 atoms lying 0.499 (3) and 0.612 (3) Å, respectively out of plane. The ring-puckering parameters are q_2_ = 0.653 (2) Å, q_3_ = -0.043 (2) Å, Q = 0.654 (2) Å, and φ_2_ = 49.76 (19)° (Cremer & Pople, 1975). The C21 substituents occupies an axial position whereas the C31, C41 and O6 substituents occupy equatorial positions. The phenyl ring occupies a position normal to the central ring as seen in the value of the dihedral angle between the two rings of 86.88 (11)°.

Two features are notable in the structure of (I). First, the crystal structure indicates the sole entrapment of the *syn* amide conformer of (I) (free acid of (II)-*syn*; Fig. 1). This is in contrast to the mixture of anti and *syn* conformers exhibited in aqueous sodium bicarbonate, in which anti conformer is favored by a ratio of 3:2 for (II) (sodium salt of (I)). The major conformer exhibited under these conditions was tentatively assigned as the anti conformer based on the stereochemistry of the major Diels-Alder product (2;S absolute configuration, Scheme 1), which is derived from the anti conformer of (II).

The crystal structure of (I) is stabilized by O–H···O and N–H···O hydrogen bonding, Table 1. Hydrogen bonds formed between the carboxylic acid-O41—H and carbonyl-O6 atoms leads to the formation of supramolecular chains aligned along the *b* axis, Fig. 3. The amide-N1—H hydrogen bonds to the carbonyl-O41 to form supramolecular chains aligned along the *c* axis, Fig. 4. Together, these hydrogen bonds consolidate molecules into a 3-D network, Fig. 5.

### Experimental

Compound (I) was prepared following a literature procedure (Lakner & Negrete, 2002). Into a 100 ml flask was added *L*-asparagine monohydrate (7.51 g, 50 mmol) and aqueous NaOH (25.0 ml; 2.0 *M*, 1.0 equiv). The mixture was stirred for 15 min and benzaldehyde (5.1 ml; 50 mmol, 1.0 equiv) was added *via* syringe over 5 min. The mixture was stirred overnight and treated with solid sodium bicarbonate (8.4 g; 2.0 equiv) followed by cooling in an ice bath to 273 K. To the vigorously stirred cold solution acryloyl chloride (5.3 ml; 65.5 mmol, 1.3 equiv) was added slowly (5 portions over 1 h). Cooling and stirring were continued an additional 2 h, after which the mixture was treated with HCl (10%, 1.6 equiv), inducing precipitation of (I). The product was filtered, washed with ice-cold water, and dried overnight under high vacuum to obtain (I) as an amorphous white powder (53% yield). The solid was crystallized twice in slow evaporating methanol to yield white crystals: *M*. pt: 457 K (dec.); [α]_D_ 24.3: -23.5° (c = 1 g cm^-3^, methanol); ^1^H NMR (sat. NaHCO_3_/D_2_O, 500 MHz; the observation of separate signals for C2—H is suggestive of two conformers): δ 2.2–2.8 (m, 2H), 4.6–4.8 (two br s, 1H), 5.93 (br d, 1H), 6.36 (d, J = 17.0 Hz, 1H), 6.60 (br s, 0.4H; signal for the minor conformer of C2—H), 6.65 (dd, J = 10.0, 17.0 Hz, 1H), 6.90 (br s, 0.6H; signal for the major conformer of C2—H) 7.4–7.8 (m, 5H). ^13^C NMR (sat. NaHCO_3_/D_2_O, 125 MHz): δ 33.5, 55.5, 126.6, 127.6, 128.7, 130.2, 138.2, 160.5, 169.2, 173.9, 176.7; IR (ν_max_, cm^-1^): 3355, 1716, 1645, 1416, 1356, 629; Anal. Calcd for C_14_H_14_N_2_O_4_: C, 61.31; H, 5.14; N, 10.21. Found: C, 60.79; H, 5.05; N, 10.10%.

Single crystals were obtained by slow evaporation of a methanol solution of (I).

### Refinement

The H atoms were geometrically placed (O—H = 0.84 Å, N–H = 0.88 Å, and C—H = 0.95–1.00 Å) and refined as riding with *U*_iso_(H) = 1.2–1.5*U*_eq_(parent atom). In the absence of significant anomalous scattering effects, 1149 Friedel pairs were averaged in the final refinement. The absolute configuration was determined on the basis of the absolute stereochemistry of (*S*)-asparagine, a reagent employed in the synthesis.

### Crystal data

**Table:**

C_14_H_14_N_2_O_4_	*F*(000) = 576
*M**_r_* = 274.27	*D*_x_ = 1.391 Mg m^−^^3^
Orthorhombic, *P*2_1_2_1_2_1_	Mo *K*α radiation, λ = 0.71069 Å
Hall symbol: P 2ac 2ab	Cell parameters from 4478 reflections
*a* = 10.573 (5) Å	θ = 1.9–30.4°
*b* = 10.670 (7) Å	µ = 0.10 mm^−^^1^
*c* = 11.612 (5) Å	*T* = 98 K
*V* = 1310.0 (12) Å^3^	Block, colourless
*Z* = 4	0.36 × 0.12 × 0.12 mm

### Data collection

**Table:**

Rigaku AFC12K/SATURN724 diffractometer	1573 independent reflections
Radiation source: fine-focus sealed tube	1547 reflections with *I* > 2σ(*I*)
graphite	*R*_int_ = 0.033
ω scans	θ_max_ = 26.5°, θ_min_ = 2.6°
Absorption correction: multi-scan (*ABSCOR*; Higashi, 1995)	*h* = −11→13
*T*_min_ = 0.496, *T*_max_ = 1	*k* = −13→13
14053 measured reflections	*l* = −13→14

### Refinement

**Table:**

Refinement on *F*^2^	Secondary atom site location: difference Fourier map
Least-squares matrix: full	Hydrogen site location: inferred from neighbouring sites
*R*[*F*^2^ > 2σ(*F*^2^)] = 0.038	H-atom parameters constrained
*wR*(*F*^2^) = 0.097	*w* = 1/[σ^2^(*F*_o_^2^) + (0.0562*P*)^2^ + 0.3429*P*] where *P* = (*F*_o_^2^ + 2*F*_c_^2^)/3
*S* = 1.11	(Δ/σ)_max_ < 0.001
1573 reflections	Δρ_max_ = 0.20 e Å^−^^3^
185 parameters	Δρ_min_ = −0.20 e Å^−^^3^
1 restraint	Extinction correction: *SHELXL97* (Sheldrick, 2008), Fc^*^=kFc[1+0.001xFc^2^λ^3^/sin(2θ)]^-1/4^
Primary atom site location: structure-invariant direct methods	Extinction coefficient: 0.009 (2)

### Special details

**Table:**

Geometry. All s.u.'s (except the s.u. in the dihedral angle between two l.s. planes) are estimated using the full covariance matrix. The cell s.u.'s are taken into account individually in the estimation of s.u.'s in distances, angles and torsion angles; correlations between s.u.'s in cell parameters are only used when they are defined by crystal symmetry. An approximate (isotropic) treatment of cell s.u.'s is used for estimating s.u.'s involving l.s. planes.
Refinement. Refinement of *F*^2^ against ALL reflections. The weighted *R*-factor *wR* and goodness of fit *S* are based on *F*^2^, conventional *R*-factors *R* are based on *F*, with *F* set to zero for negative *F*^2^. The threshold expression of *F*^2^ > 2σ(*F*^2^) is used only for calculating *R*-factors(gt) *etc*. and is not relevant to the choice of reflections for refinement. *R*-factors based on *F*^2^ are statistically about twice as large as those based on *F*, and *R*- factors based on ALL data will be even larger.

### Fractional atomic coordinates and isotropic or equivalent isotropic displacement parameters (Å^2^)

**Table:**

	*x*	*y*	*z*	*U*_iso_*/*U*_eq_	
O6	0.81404 (14)	0.17595 (13)	0.43043 (13)	0.0272 (4)	
O31	0.97062 (14)	0.69803 (15)	0.42240 (13)	0.0280 (4)	
O41	0.90743 (14)	0.61359 (15)	0.17482 (13)	0.0292 (4)	
O42	1.09113 (14)	0.52210 (15)	0.21965 (13)	0.0281 (4)	
H42o	1.1171	0.5703	0.1676	0.042*	
N1	0.73021 (17)	0.36445 (17)	0.46856 (15)	0.0242 (4)	
H1	0.7003	0.3381	0.5350	0.029*	
N3	0.82135 (16)	0.55157 (16)	0.39712 (15)	0.0230 (4)	
C4	0.91724 (19)	0.4760 (2)	0.33983 (17)	0.0228 (4)	
H4	0.9877	0.4602	0.3954	0.027*	
C5	0.8624 (2)	0.3497 (2)	0.30244 (18)	0.0243 (4)	
H5A	0.7995	0.3626	0.2404	0.029*	
H5B	0.9307	0.2955	0.2721	0.029*	
C6	0.80012 (19)	0.2871 (2)	0.40449 (18)	0.0238 (4)	
C2	0.70244 (19)	0.49147 (19)	0.43056 (18)	0.0232 (4)	
H2	0.6679	0.5384	0.4982	0.028*	
C21	0.60132 (19)	0.49244 (19)	0.33662 (18)	0.0245 (4)	
C22	0.6093 (2)	0.5707 (2)	0.24203 (19)	0.0297 (5)	
H22	0.6795	0.6254	0.2335	0.036*	
C23	0.5135 (2)	0.5692 (3)	0.1587 (2)	0.0337 (5)	
H23	0.5192	0.6231	0.0939	0.040*	
C24	0.4116 (2)	0.4907 (2)	0.1699 (2)	0.0361 (6)	
H24	0.3479	0.4888	0.1122	0.043*	
C25	0.4020 (2)	0.4142 (2)	0.2656 (3)	0.0435 (7)	
H25	0.3308	0.3608	0.2745	0.052*	
C26	0.4962 (2)	0.4155 (2)	0.3487 (3)	0.0372 (6)	
H26	0.4887	0.3632	0.4146	0.045*	
C31	0.86129 (19)	0.6649 (2)	0.43839 (18)	0.0236 (4)	
C32	0.7670 (2)	0.7439 (2)	0.4996 (2)	0.0274 (5)	
H32	0.6795	0.7333	0.4839	0.033*	
C33	0.8035 (2)	0.8280 (2)	0.5748 (2)	0.0380 (6)	
H33A	0.8909	0.8389	0.5907	0.046*	
H33B	0.7425	0.8780	0.6134	0.046*	
C41	0.97069 (19)	0.5461 (2)	0.23591 (17)	0.0232 (4)	

### Atomic displacement parameters (Å^2^)

**Table:**

	*U*^11^	*U*^22^	*U*^33^	*U*^12^	*U*^13^	*U*^23^
O6	0.0262 (8)	0.0274 (7)	0.0281 (7)	0.0023 (6)	0.0043 (6)	0.0022 (6)
O31	0.0197 (7)	0.0332 (8)	0.0311 (8)	−0.0026 (6)	−0.0005 (6)	−0.0024 (7)
O41	0.0213 (7)	0.0410 (9)	0.0253 (7)	−0.0003 (7)	−0.0024 (6)	0.0056 (7)
O42	0.0215 (7)	0.0319 (8)	0.0310 (8)	0.0003 (7)	0.0069 (7)	0.0045 (7)
N1	0.0233 (9)	0.0262 (8)	0.0229 (8)	0.0022 (7)	0.0041 (7)	0.0026 (7)
N3	0.0168 (8)	0.0277 (9)	0.0245 (8)	−0.0006 (7)	0.0022 (7)	−0.0029 (7)
C4	0.0172 (9)	0.0279 (10)	0.0232 (9)	−0.0008 (8)	0.0011 (8)	−0.0005 (8)
C5	0.0215 (10)	0.0274 (10)	0.0242 (10)	0.0014 (8)	0.0019 (8)	−0.0026 (8)
C6	0.0170 (9)	0.0300 (10)	0.0243 (10)	−0.0009 (8)	−0.0011 (8)	−0.0016 (8)
C2	0.0197 (10)	0.0250 (9)	0.0248 (9)	0.0010 (8)	0.0022 (8)	−0.0010 (8)
C21	0.0203 (10)	0.0247 (10)	0.0285 (10)	0.0029 (8)	0.0022 (9)	−0.0036 (8)
C22	0.0208 (10)	0.0396 (12)	0.0287 (10)	−0.0018 (9)	0.0014 (8)	0.0021 (10)
C23	0.0254 (11)	0.0457 (13)	0.0301 (11)	0.0046 (10)	−0.0002 (9)	0.0004 (11)
C24	0.0241 (11)	0.0404 (13)	0.0438 (13)	0.0063 (10)	−0.0094 (11)	−0.0102 (11)
C25	0.0276 (12)	0.0316 (12)	0.0714 (19)	−0.0058 (10)	−0.0108 (13)	0.0022 (13)
C26	0.0246 (12)	0.0322 (11)	0.0549 (15)	−0.0031 (10)	−0.0044 (11)	0.0088 (11)
C31	0.0198 (10)	0.0279 (10)	0.0231 (10)	−0.0007 (8)	−0.0013 (8)	−0.0009 (8)
C32	0.0223 (10)	0.0287 (10)	0.0310 (10)	−0.0010 (9)	0.0032 (8)	−0.0009 (9)
C33	0.0289 (12)	0.0407 (13)	0.0445 (14)	−0.0019 (10)	0.0046 (11)	−0.0134 (11)
C41	0.0178 (10)	0.0288 (10)	0.0230 (9)	−0.0025 (8)	−0.0001 (8)	−0.0037 (8)

### Geometric parameters (Å, °)

**Table:**

O6—C6	1.233 (3)	C2—H2	1.0000
O31—C31	1.223 (3)	C21—C22	1.382 (3)
O41—C41	1.212 (3)	C21—C26	1.389 (3)
O42—C41	1.313 (3)	C22—C23	1.400 (3)
O42—H42o	0.8400	C22—H22	0.9500
N1—C6	1.334 (3)	C23—C24	1.371 (3)
N1—C2	1.455 (3)	C23—H23	0.9500
N1—H1	0.8800	C24—C25	1.383 (4)
N3—C31	1.367 (3)	C24—H24	0.9500
N3—C4	1.456 (3)	C25—C26	1.387 (4)
N3—C2	1.464 (3)	C25—H25	0.9500
C4—C41	1.528 (3)	C26—H26	0.9500
C4—C5	1.530 (3)	C31—C32	1.486 (3)
C4—H4	1.0000	C32—C33	1.310 (3)
C5—C6	1.511 (3)	C32—H32	0.9500
C5—H5A	0.9900	C33—H33A	0.9500
C5—H5B	0.9900	C33—H33B	0.9500
C2—C21	1.527 (3)		
			
C41—O42—H42o	107.5	C22—C21—C2	121.94 (19)
C6—N1—C2	121.25 (18)	C26—C21—C2	118.9 (2)
C6—N1—H1	119.4	C21—C22—C23	119.9 (2)
C2—N1—H1	119.4	C21—C22—H22	120.1
C31—N3—C4	115.77 (17)	C23—C22—H22	120.1
C31—N3—C2	124.03 (17)	C24—C23—C22	120.6 (2)
C4—N3—C2	118.46 (16)	C24—C23—H23	119.7
N3—C4—C41	110.29 (17)	C22—C23—H23	119.7
N3—C4—C5	110.71 (16)	C23—C24—C25	119.6 (2)
C41—C4—C5	110.33 (17)	C23—C24—H24	120.2
N3—C4—H4	108.5	C25—C24—H24	120.2
C41—C4—H4	108.5	C24—C25—C26	120.1 (2)
C5—C4—H4	108.5	C24—C25—H25	120.0
C6—C5—C4	109.38 (17)	C26—C25—H25	120.0
C6—C5—H5A	109.8	C25—C26—C21	120.7 (2)
C4—C5—H5A	109.8	C25—C26—H26	119.7
C6—C5—H5B	109.8	C21—C26—H26	119.7
C4—C5—H5B	109.8	O31—C31—N3	119.6 (2)
H5A—C5—H5B	108.2	O31—C31—C32	122.9 (2)
O6—C6—N1	121.7 (2)	N3—C31—C32	117.51 (19)
O6—C6—C5	124.39 (19)	C33—C32—C31	120.7 (2)
N1—C6—C5	113.90 (18)	C33—C32—H32	119.7
N1—C2—N3	108.37 (16)	C31—C32—H32	119.7
N1—C2—C21	111.35 (16)	C32—C33—H33A	120.0
N3—C2—C21	114.12 (17)	C32—C33—H33B	120.0
N1—C2—H2	107.6	H33A—C33—H33B	120.0
N3—C2—H2	107.6	O41—C41—O42	124.6 (2)
C21—C2—H2	107.6	O41—C41—C4	123.30 (19)
C22—C21—C26	119.1 (2)	O42—C41—C4	112.13 (18)
			
C31—N3—C4—C41	−60.1 (2)	N3—C2—C21—C26	166.10 (19)
C2—N3—C4—C41	134.34 (18)	C26—C21—C22—C23	−1.6 (3)
C31—N3—C4—C5	177.53 (17)	C2—C21—C22—C23	−179.3 (2)
C2—N3—C4—C5	11.9 (2)	C21—C22—C23—C24	0.0 (4)
N3—C4—C5—C6	−52.8 (2)	C22—C23—C24—C25	1.4 (4)
C41—C4—C5—C6	−175.13 (16)	C23—C24—C25—C26	−1.2 (4)
C2—N1—C6—O6	−172.90 (19)	C24—C25—C26—C21	−0.5 (4)
C2—N1—C6—C5	9.2 (3)	C22—C21—C26—C25	1.8 (4)
C4—C5—C6—O6	−134.5 (2)	C2—C21—C26—C25	179.6 (2)
C4—C5—C6—N1	43.3 (2)	C4—N3—C31—O31	2.4 (3)
C6—N1—C2—N3	−50.5 (2)	C2—N3—C31—O31	167.06 (19)
C6—N1—C2—C21	75.8 (2)	C4—N3—C31—C32	−178.79 (18)
C31—N3—C2—N1	−127.48 (19)	C2—N3—C31—C32	−14.1 (3)
C4—N3—C2—N1	36.8 (2)	O31—C31—C32—C33	−25.7 (4)
C31—N3—C2—C21	107.8 (2)	N3—C31—C32—C33	155.5 (2)
C4—N3—C2—C21	−87.9 (2)	N3—C4—C41—O41	−35.8 (3)
N1—C2—C21—C22	−139.2 (2)	C5—C4—C41—O41	86.9 (2)
N3—C2—C21—C22	−16.2 (3)	N3—C4—C41—O42	145.80 (17)
N1—C2—C21—C26	43.0 (3)	C5—C4—C41—O42	−91.6 (2)

### Hydrogen-bond geometry (Å, °)

**Table:**

*D*—H···*A*	*D*—H	H···*A*	*D*···*A*	*D*—H···*A*
N1—H1···O41^i^	0.88	2.05	2.812 (3)	144
O42—H42o···O6^ii^	0.84	1.76	2.596 (3)	173

Symmetry codes: (i) −*x*+3/2, −*y*+1, *z*+1/2; (ii) −*x*+2, *y*+1/2, −*z*+1/2.
